# Shifts in diversity and function of lake bacterial communities upon glacier retreat

**DOI:** 10.1038/ismej.2015.245

**Published:** 2016-01-15

**Authors:** Hannes Peter, Ruben Sommaruga

**Affiliations:** 1 University of Innsbruck, Institute of Ecology, Lake and Glacier Research Group, Technikerstr. 25, Innsbruck, Austria

## Abstract

Global climate change is causing a wastage of glaciers and threatening biodiversity in glacier-fed ecosystems. The high turbidity typically found in those ecosystems, which is caused by inorganic particles and result of the erosive activity of glaciers is a key environmental factor influencing temperature and light availability, as well as other factors in the water column. Once these lakes loose hydrological connectivity to glaciers and turn clear, the accompanying environmental changes could represent a potential bottleneck for the established local diversity with yet unknown functional consequences. Here, we study three lakes situated along a turbidity gradient as well as one clear unconnected lake and evaluate seasonal changes in their bacterial community composition and diversity. Further, we assess potential consequences for community functioning. Glacier runoff represented a diverse source community for the lakes and several taxa were able to colonize downstream turbid habitats, although they were not found in the clear lake. Operational taxonomic unit-based alpha diversity and phylogenetic diversity decreased along the turbidity gradient, but metabolic functional diversity was negatively related to turbidity. No evidence for multifunctional redundancy, which may allow communities to maintain functioning upon alterations in diversity, was found. Our study gives a first view on how glacier-fed lake bacterial communities are affected by the melting of glaciers and indicates that diversity and community composition significantly change when hydrological connectivity to the glacier is lost and lakes turn clear.

## Introduction

Glaciers respond to alterations in both, temperature and precipitation (Oerlemans, 2005), and the global retreat of glaciers and ice sheets has become one of the most prominent signals of anthropogenic climate change (Vaughan *et al.*, 2013). The presence or absence of glaciers in catchments dominates the abiotic conditions in high mountain freshwater ecosystems (Jacobsen *et al.*, 2012; Freimann *et al.*, 2014). For instance, glaciers are a source of carbon (Hood *et al.*, 2009; Singer *et al.*, 2012; Stubbins *et al.*, 2012) and nutrients (Slemmons and Saros, 2012; Sommaruga, 2015). Further, glacier basal motion causes abrasion of bedrock and particle formation. Meltwater transports these fine inorganic particles, leading to high turbidity in glacier-fed (that is, proglacial) freshwater ecosystems (Egholm *et al.*, 2012). These particles range in size from clay to silt and pose a real challenge for filter-feeding organisms such as *Daphnia* or phagotrophic nanoflagellates (Koenings *et al.*, 1990; Sommaruga and Kandolf, 2014; Sommaruga, 2015). However, turbidity drives other relevant environmental factors such as temperature (Gallegos *et al.*, 2008) and light availability in the water column (Rose *et al.*, 2014), causing unfavorable conditions for primary producers. In comparison with clear high mountain lakes, the communities of turbid proglacial lakes may thus be dominated by heterotrophic and eventually mixotrophic microbes (Sommaruga, 2015).

Glacier retreat has been shown to threaten the specialized local biodiversity of macroinvertebrates (Milner *et al.*, 2009; Jacobsen *et al.*, 2012) and microorganisms in proglacial stream and floodplain ecosystems (Freimann *et al.*, 2013; Wilhelm *et al.*, 2013), but the consequence for the functioning of these ecosystems remains unresolved. On the short term, glacier retreat may increase turbidity in proglacial lakes, however, as glaciers continue to melt, the contribution of glacier meltwater to proglacial lakes will diminish. In the European Alps, for instance, wastage of glacier is most significant below 3000 m above sea level, and low-altitude glaciers are predicted to disappear within this century (Zemp *et al.*, 2009). As the complete disappearance of glaciers will markedly influence the physical conditions of proglacial lakes, biodiversity and ecological functioning are likely to be affected by such a regime shift (Scheffer, 2003).

Headwater systems in general act as reservoirs of microbial diversity (Besemer *et al.*, 2013) and liquid environments on and within glaciers are now recognized as habitat to diverse and active microbial assemblages (Anesio and Laybourn-Parry, 2012). Although glacier-derived microbial diversity has recently been shown to contribute only marginally to proglacial stream and biofilm communities (Wilhelm *et al.*, 2013), the role of glacier runoff as a source of diversity for glacier-fed lakes remains unclear.

Here, we present results from a field study of four lakes in the proximity of a rapidly receding glacier in the Austrian Alps. Three of the lakes are connected to the glacier and situated along an altitudinal transect and represent a turbidity gradient. The fourth lake, situated behind a rocky ridge, has lost contact to the glacier and is clear. All lakes are remote and mostly unaffected by direct anthropogenic disturbance, highlighting their sentinel role in the landscape (Adrian *et al.*, 2009).

We study the effects of glacial turbidity on bacterial community composition and diversity and estimate potential functional consequences using metagenome prediction. Furthermore, we assess the role of glacier runoff as a source of diversity for this metacommunity using a Bayesian mixing model. We hypothesize that bacterial diversity increases with decreasing turbidity, reflecting the contribution of allochthonous resources to glacier runoff. However, owing to limiting light availability and colder water temperature (Slemmons *et al.*, 2013; Rose *et al.*, 2014), we expect to find overall reduced diversity compared with the clear lake. We further hypothesize that the shift from turbid to clear conditions represents a bottleneck for the microbial communities that colonized the glacier-fed lakes, which likely is accompanied by a shift in community functioning.

## Materials and methods

### Study site and sampling

The Faselfad lakes are situated in the Austrian Central Alps and originated from a single, rapidly retreating glacier, which is located on a steep slope (for overview maps and photographs, see Supplementary Material 1). The lakes are fish- and cladoceran-free (Sommaruga and Kandolf, 2014; Kammerlander *et al.*, 2015) and were sampled four times during the ice-free season in 2012 (Supplementary Table 1). The lakes are situated along an altitudinal gradient. The uppermost and most turbid lakes (FAS 1) is located close to the glacier terminus at 2.620 m a.s.l. Approximately 200 m of altitude lower, the two turbid lakes FAS 2 and FAS 3 are most of the year connected and therefore, we sampled only the deeper and larger FAS 3. At the same altitude of *ca*. Two thousand four hundred m a.s.l., FAS 4 is located behind a rocky ridge. The glacier has already receded beyond the ridge and FAS 4 has since become clear. A shallow (~2 m deep) pond (FAS 5) is located close to FAS 4, however, since this pond entirely freezes during winter, we did not sample it. Finally, water from the turbid lake FAS 3 and from the clear lake FAS 4 mixes in the lowest lake FAS 6. There is no distinct proglacial stream at the glacier terminus, rather the glacier meltwater flows through barren substrate below the glacier terminus. Glacier runoff was collected before entering into the uppermost lake (FAS 1) and therefore, it not only will contain microbes from glacier ice meltwater, but also from the sediments adjacent to the glacier terminus. These remote sites were reached by helicopter and sampled on two consecutive days. Three replicated composite samples from the whole water column (that is, same volume pooled from single depths) were taken with a 5 l water sampler from a boat anchored above the deepest point. The samples were stored in 10 l carboys at *in situ* temperatures, protected from solar radiation by a dark plastic foil, and transported immediately after sampling to the laboratory. Upon arrival, 750 ml were filtered onto 0.2 μm polyethersulfone filters (GPWP, Merck Millipore, Billerica, MA, USA), and stored at −80 °C for later molecular analyses.

### Environmental parameter

Nephelometric turbidity of composite water samples was measured using a portable instrument (Turb 430 T, WTW, Weilheim, Germany), which measures 90° scattered ‘white' light (Tungsten lamp). This measurement is tailored to measure turbidity caused by small inorganic particles. Samples for water chemistry analyses were filtered through glass-fiber filter (GF/F, Whatman, Maidstone, VT, USA). The concentration of total dissolved phosphorus was determined spectrophotometrically using the molybdate method after digestion with sulfuric acid and hydrogen peroxide. Samples for dissolved organic carbon (non-purgeable organic carbon) and dissolved nitrogen measurements were acidified to pH 2 and analyzed (within 48 h) with a total organic carbon analyzer (Shimadzu TOC-Vc series, Kyoto, Japan) equipped with a total nitrogen module.

### DNA extraction and sequencing

Bacterial community composition was analyzed using parallel sequencing of the V4 region of the 16S ribosomal RNA gene on the 454 GS FLX platform with Titanium chemistry (Roche, Basel, Switzerland). DNA was extracted using the PowerWater DNA extraction kit (Mobio, Carlsbad, CA, USA). Triplicated PCR reactions using the barcode–primer combinations described in Fierer *et al.* (2008), purification, quantification and equimolar mixing of the samples before sequencing were done by EnGencore (Greenville, SC, USA). In total, 1 161 057 sequences were obtained. Bioinformatic processing of sequences was performed in mothur following the standard operational protocol (Schloss *et al.*, 2011), including PyroNoise, which reduces the sequencing error rate by correcting the original flowgram data (Schloss *et al.*, 2011). Sequences were aligned against the SILVA reference database and taxonomically assigned using mothur's naive Bayesian classifier. Sequences identified as Eukaryota, Archaea, Chloroplasts, Mitochondria or unknown phlya were removed. Chimeric sequences were removed using uchime. Pairwise sequence distances were calculated treating gaps of any length as single insertions and sequences were clustered into operational taxonomic units at the 97% similarity level. After denoising, 667 312 sequences remained, which clustered into 10 163 operational taxonomic units (OTUs). The sequence data have been submitted to the NCBI Sequence Read Archive under BioProject ID PRJNA297573.

### Multivariate statistical analyses and diversity estimates

Community data sets were rarified to 2421 individuals and 7 samples with fewer sequences were removed from further analyses. Bootstrap OTU richness, the abundance-based coverage estimator and nonparametric Shannon evenness were calculated using mothur. Multivariate and statistical analyses were performed using the statistical environment R (R Development Core Team, 2013) and the packages vegan (Oksanen *et al.*, 2013), GUniFrac (Chen *et al.*, 2012) and picante (Kembel *et al.*, 2010). Vegan's functions *metaMDS* was used for ordinations, *envfit* was used to fit environmental vectors and *ordisurf*, which uses generalized additive models, was used to visualize the gradient of turbidity in the ordinations. Function *adonis* was used to test for significance between lakes in the ordinations. Weighted generalized UniFrac distance was calculated with alpha (controlling the weight of abundant lineages) set to 0.5. Faith's phylogenetic diversity (PD) is the number of OTUs in a sample multiplied by average branch length of the phylogenetic tree. The phylogenetic tree used for PD and generalized Unifrac distance was calculated using FastTree vers. 2.1.7 (Price *et al.*, 2010), applying the generalized time-reversible model.

### Identifying characteristic community members

To identify phylogenetically coherent, characteristic members of communities along the turbidity gradient, we applied the LEfSe method (Segata *et al.*, 2011). LEfSe first uses nonparametric factorial Kruskal–Wallis sum rank tests (alpha=0.01) to detect differential abundant features (that is, genera, families, classes, phyla) in three categories along the turbidity gradient (clear: 0–0.3 NTU *n*=4; intermediate: 1.3–6.3 NTU *n*=7, turbid: 9.9–42.8 NTU *n*= 5). LEfSe then tests the phylogenetic consistency using pairwise Wilcoxon rank-sum tests (alpha=0.01), and finally estimates effect size of each differentially abundant feature using linear discriminant analysis. All-against-all classes were compared (most stringent) and a value of 2.0 of the logarithmic linear discriminant analysis score was chosen as threshold for discriminative features.

### Sources of bacterial diversity

SourceTracker, a Bayesian mixing model (Knights *et al.*, 2011) was used to identify potential sources among the proglacial lake bacterial assemblages using default settings and *α* set to 0.001. The glacier runoff and FAS 1 were used as potential source communities for the downstream turbid (FAS 3 and FAS 6) and the unconnected clear lake FAS 4. FAS 1 was included as a potential source in order to estimate the regional pool of freshwater bacteria in comparison with the glacier runoff community.

### Functional gene prediction and the estimation of multifunctional redundancy

Functional changes along the turbidity gradient were assessed using Phylogenetic Investigation of Communities by Reconstruction of Unobserved States (PICRUSt) (Langille *et al.*, 2013). PICRUSt is a bioinformatics tool, which allows for the reconstruction of a metagenome by inference of gene content using 16S ribosomal DNA sequences. For this, PICRUSt uses full genome sequenced relatives of the OTUs and ancestral state reconstruction. Briefly, the rarefied 16S ribosomal DNA sequences are aligned with the Greengenes database (vers. 13.5) (DeSantis *et al.*, 2006), then the PICRUSt algorithm adjusts for 16S gene copy number, and finally predicts functional genes, which are further classified into KEGG (Kyoto Encyclopedia of Genes and Genomes) orthologues. We used the predicted gene category abundances and calculated multifunctional diversity using Shannon H as a diversity index. Thus, communities with a high predicted multifunctional diversity harbor many different and evenly distributed gene categories, whereas a low multifunctional diversity indicates fewer gene categories or the dominance of a few gene categories in a community. Predicted multifunctional diversity was related to turbidity and used according the framework introduced by Miki *et al.* (2013) to estimate multifunctional redundancy. For this, multifunctional diversity is related to taxa richness (here we used Shannon H for OTUs and PD accounting for phylogenetic relatedness of the OTUs). The shape of the relationship is indicative for the degree of multifunctional redundancy within a metacommunity. A linear relationship indicates that with increasing taxa diversity, multifunctional diversity increases too, whereas a saturating relationship indicates functional redundancy at the metacommunity level.

## Results and discussion

### Changes in bacterial community composition along the turbidity gradient

Turbidity in glacier-fed lakes exhibited pronounced seasonal variation, ranging between 1.3 and 13.3 NTU at the beginning of the ice-free season, when snowmelt dominated runoff. Turbidity peaked in mid-summer, reaching between 6.3 and 42.8 NTU (Supplementary Table 1). Total dissolved phosphorus was significantly higher in glacier-fed lakes than in the clear lake (bootstrapped *t*-test: *t*=2.59, *P*=0.02) and tracked seasonal changes in turbidity in glacier-fed lakes (linear regression, *R*^2^=0.62, *P*=0.001), which reflects the release of inorganic P from the glacier bedrock in this catchment. The release of nutrients (for example, iron; Arrigo *et al.*, 2015) by glacier erosive activity has been shown to influence recipient ecosystems, however, the role of glacier-derived inorganic nutrients in high mountain ecosystems remains unknown. Dissolved nitrogen concentration did not show pronounced seasonal trends or significant differences between clear and turbid lakes (bootstrapped *t*-test: *t*=0.79, *P*=0.44), which contrasts with glacier-influenced freshwater systems in North America (Hood and Berner, 2009; Slemmons and Saros, 2012).

As turbidity scales with glacier coverage (Hood and Berner, 2009), we used turbidity as a proxy for the influence of glacier runoff on proglacial lakes. In fact, dissolved organic carbon concentration paralleled seasonal changes in turbidity, which may reflect the release of carbon (partly black carbon from fossil fuel combustion) stored in glacier ice (Hood *et al.*, 2009; Singer *et al.*, 2012; Stubbins *et al.*, 2012). However, DOC concentrations were not significantly different between the clear and turbid lakes (bootstrapped *t*-test: *t*=0.94, *P*=0.36) and since the unconnected clear lake also showed a peak in DOC concentration in mid-summer, autochthonous and allochthonous sources likely contribute to seasonality of DOC in this catchment. This notion is further supported by relatively high concentrations of chlorophyll-*a* found in all lakes in mid-summer (Supplementary Table 1). Thus, key environmental factors in glacier-fed lakes co-vary over the course of the ice-free season, reflecting the contribution of glacier meltwater and seepage water to local hydrology.

**Figure 1:**
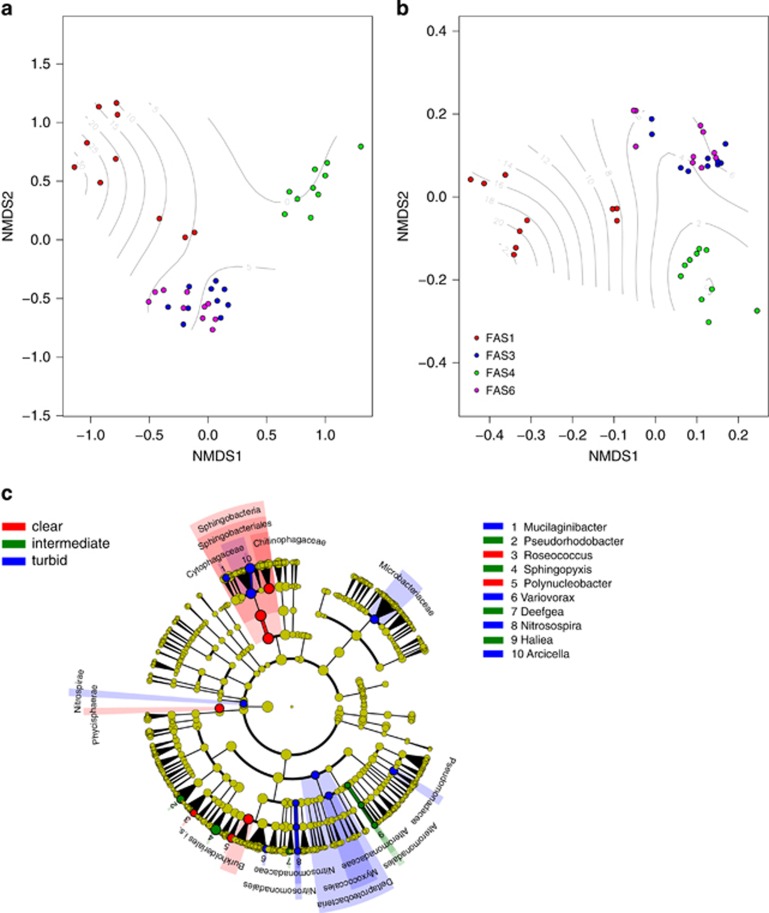
Bacterial community compositional similarity. Shown are non-metric multidimensional scaling plots based on Bray–Curtis dissimilarity (**a**) and the weighted generalized Unifrac distance reflecting phylogenetic relatedness (**b**). The filled circles reflect bacterial community composition in the different lakes, with colors referring to the turbid lakes FAS 1, FAS 3 and FAS 6 and the clear lake FAS 4 according to the legend. Contour lines show a smooth general additive model surface reflecting the turbidity gradient among the samples. The cladogram (**c**) visualizes the output of the LEfSe algorithm, which identifies taxonomically consistent differences between clear (0–0.3 NTU), intermediate (1.3–6.3 NTU) and turbid (9.9–42.8 NTU) community members. Taxa with nonsignificant differences are represented as yellow circles and the diameter of the circles are proportional to relative abundance.

Bacterial community composition was significantly structured along the turbidity gradient (Figure 1, environmental factor fit: *R*^2^=0.46, *P*=0.001). Analysis of variance using distance matrices indicated significant differences in community structure among the lakes, both based on the abundance of operational taxonomic units (Bray–Curtis dissimilarity; *R*^2^=0.47, *P*=0.001) and on phylogenetic distance between community members (generalized Unifrac distance; *R*^2^=0.67, *P*=0.001). Work on hyporheic stream sediments in glaciated floodplains has revealed an influence of glacier meltwater on the structure and function of microbial communities (Freimann *et al.*, 2013). However, functional redundancy along with subtle shifts in community composition impeded the quantification of functional consequences of glacier retreat in these systems (Freimann *et al.*, 2013). In glacier-fed lakes, turbidity co-varies with environmental conditions such as temperature, nutrients, light and ultraviolet (UV) radiation, as well as with biotic factors such as primary production and the relative abundance of grazers. Therefore, teasing apart these factors and understanding the mechanisms of community assembly and dynamics will need experimental work (Sommaruga, 2015).

### Characteristic community members

Bacterial communities in the Faselfad lakes were generally characterized by Bacteroidetes related to Sphingobacteria (on average 23%) and Flavobacteria (on average 14%) and betaproteobacterial taxa (on average 19%). The most abundant OTUs were also found in most samples (Supplementary Figure 1C). Interestingly, there was virtually no diversity exclusively shared between the glacier runoff and the clear lake (Supplementary Figure 1A), whereas several OTUs related to Betaproteobacteria (for example, *Polaromonas*, *Methylotenera*, *Albidiferax* and *Curvibacter*) occurred in the glacier runoff and the turbid lakes, but were absent in the clear lake (Supplementary Figure 1D). In contrast, the communities in the clear lake exclusively contained several OTUs related to Sphingobacteria (for example, *Haliscomenobacter*, unclassified Cytophagaceae and Chitinophagaceae, *Polynucleobacter* and *Ferruginibacter*).

Twenty-five phylogenetic consistent units were identified as statistically significant discriminative either for clear (*n*=7), intermediate (*n*=6) or turbid (*n*=12) lake communities (Figure 1c). At the phylum level, Nitrospirae were the only group characteristic for turbid conditions. Chemolithotrophic Nitrospirae have previously been detected in glacier runoff (Wilhelm *et al.*, 2013), are common in proglacial soils (Supplementary Table 2), and have probably important functions in proglacial lakes. Sphingobacteria (for example, *Arcicella*, *Mucilaginibacter*), Betaproteobacteria of the family Nitrosomonadales (for example, *Nitrospira*) and Deltaproteobacteria further characterized turbid lake communities. Betaproteobacteria of the family Burkholeriales (for example, *Polynucleobacter*) and Alphaproteobacteria (for example, *Roseococcus*) were distinctive features of the clear lake communities. Gammaproteobacteria of the family Alteromonadales (for example, *Haliea*), Betaproteobacteria of the family Neisseriales (for example, *Deefgea*) and Alphaproteobacteria (for example, *Sphingopyxis*, *Pseudorhodobacter*) were characteristic community members of lakes of intermediate turbidity.

### Alpha diversity

Out of 10 163 OTUs, most OTUs occurred exclusively in either one of the turbid lakes (*n*=5856, 57.6%) or the glacier runoff (*n*=2220, 21.8%), whereas only 9% of all OTUs (*n*=916) were exclusively detected in the clear lake or shared among the different systems (*n*= 1171, 11% Supplementary Figure 1B). Abundant OTUs, however, were found in most samples (Supplementary Figure 1C).

**Figure 2:**
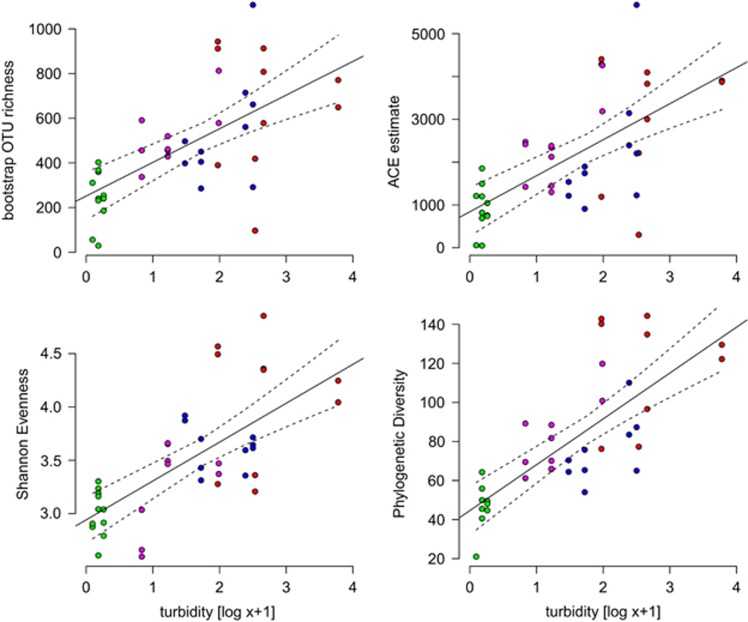
Indices used to describe alpha diversity of bacterial assemblages in lakes along a turbidity gradient (colors same as in Figure 1). Shown are bootstrapped OTU richness, abundance-based coverage estimate (ACE), the nonparametric Shannon evenness index, and Faith's PD. Colors are the same as in Figure 1. Solid lines indicate linear model fits between turbidity (log x+1) and the respective alpha diversity estimate. Dashed lines represent the 95% confidence intervals of the model fits.

Alpha diversity estimates differed significantly among the different lakes, such as the bootstrap number of OTUs (Welch F=12.78, *P*<0.01) and the abundance-based coverage estimate, an abundance-based estimate of OTU richness (Welch F=10.24, *P*<0.01; Supplementary Figure 2). Significant differences among lakes were also found in nonparametric Shannon evenness, which is low in communities dominated by few taxa and high in more diverse communities with taxa of similar abundance (Welch F=16.27, *P*<0.01) and in PD, which is a measure of diversity adjusted for the relatedness of community members (Welch F=8.66, *P*<0.01). All indices revealed a great bacterial diversity in the glacier runoff, however, these assemblages were also characterized by a large evenness, with only very few taxa being numerically dominant (Supplementary Figure 3). The turbid lakes featured reduced diversities compared with the glacier runoff, both in OTU richness and PD. Lowest alpha diversities were detected in the clear lake FAS 4, consistently throughout the season. Alpha diversity estimates, thus, exhibited positive trends along the turbidity gradient in the Faselfad lakes (Figure 2). The bootstrapped number OTUs and abundance-based coverage estimates were positively related to turbidity (linear regression: *R*^2^=0.38, *P*=0.001 and *R*^2^=0.41, *P*=0.001, respectively). Nonparametric Shannon evenness and PD were positively related to turbidity (linear regression: *R*^2^=0.47, *P*=0.001 and *R*^2^=0.42, *P*=0.001, respectively). A higher microbial diversity in glacier-fed lakes than in the clear lake seems surprising, given the cold and light-limited environment. However, Wilhelm *et al.* (2013), reported diverse and dynamic communities in proglacial high mountain streams, with alpha diversity estimates ranging between 500 and 4500 taxa in the runoff of 26 Alpine glaciers. Liquid environments on, within and below glacier ice masses are now recognized as habitat to complex microbial communities (Anesio and Laybourn-Parry, 2012), and members of glacier communities may augment proglacial lake communities. Similarly, Crump *et al.* (2012) reported a downstream gradient in bacterial alpha diversity in arctic freshwater systems. They suggest that dispersal, that is, ‘mass effects' in metacommunity theory (Leibold *et al.*, 2004), dominates over local extinction in freshwater systems of short residence time. This may also be true for high mountain lakes, however, would not explain the difference between turbid and clear lakes. Instead, we argue that the contrasting environmental conditions observed along the turbidity gradient shape bacterial diversity. For instance, the absence or reduced abundance of key predators such as heterotrophic nanoflagellates in turbid glacial lakes (Sommaruga and Kandolf, 2014) could be an important additional driving force for differences in diversity. In the same line, because solar UV radiation is rapidly attenuated by suspended particles in glacier-fed lakes (Rose *et al.*, 2014), turbid lakes may provide a refuge to UV-sensitive taxa and sustain UV-sensitive ecosystem functions.

### Sources of diversity

**Figure 3:**
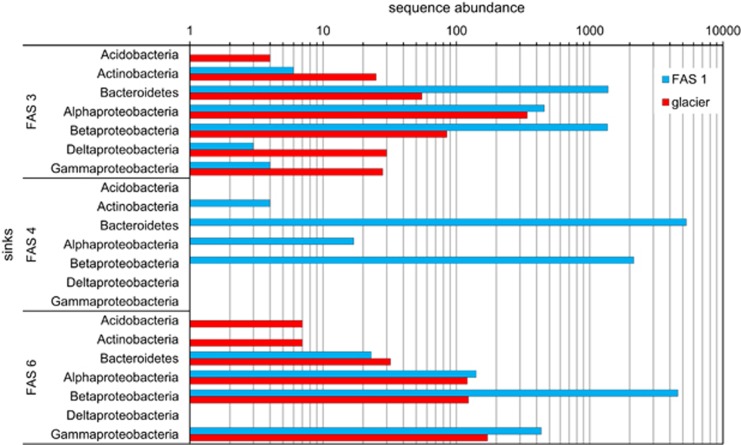
Results of the Bayesian mixing model using the glacier runoff and the uppermost turbid lake FAS 1 as sources to the connected downstream turbid lakes FAS 3 and FAS 6 and the unconnected clear lake FAS 4 (sinks). Bars represent sequence abundance binned by phylogenetic class affiliation for significant taxa.

Using a Bayesian mixing model (Sourcetracker), we evaluated the importance of glacier runoff as a source of diversity for downstream lake ecosystems (Figure 3). As a reference, we included the uppermost turbid lake as another source, which in addition to the glacier-derived communities may harbor a regional freshwater species pool. Although a number of bacterial taxa were identified to be glacier derived in the turbid lakes, not a single bacterial OTU was detected to be so in the unconnected, clear lake. The most abundant glacier-derived bacterial classes included Alpha- and Betaproteobacteria in both downstream turbid lakes. Glacier-derived Deltaproteobacteria were found in FAS 3, but not in FAS 6, however, Gammaproteobacteria showed the opposite pattern, with the glacier runoff being a significant source for FAS 6. In addition to the glacier as a potential source of diversity, a surprisingly large regional species pool seems to exist in this high mountain environment, as indicated by the number of Bacteroidetes and Betaproteobacteria, which potentially colonized the unconnected clear lake. Bacteroidetes can express grazing resistant morphologies (Salcher *et al.*, 2005), and it remains to be tested whether their ubiquity along the turbidity gradient is related to this trait. Moreover, microbes in glaciers are probably dispersed from close and distant locations, often associated with dispersal vectors such as terrestrial dust or aerosols (Margesin and Miteva, 2011). Once deposited with snow, they are gradually embedded in deep ice layers and released upon melting. Hence, the ongoing melting of glaciers will continue to release a diversity of microorganisms to downstream environments.

### Potential functional consequences of glacier retreat

**Figure 4:**
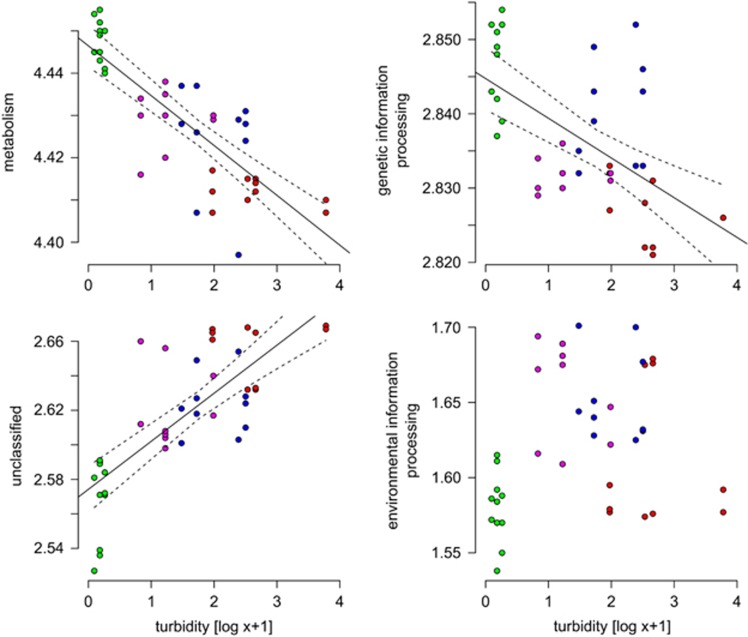
Multifunctional diversity (Shannon diversity index) of the most abundant KEGG functions classified as metabolism, genetic information processing, environmental information processing and unclassified traits as a function of turbidity. Color codes are the same as in Figure 1.

**Figure 5:**
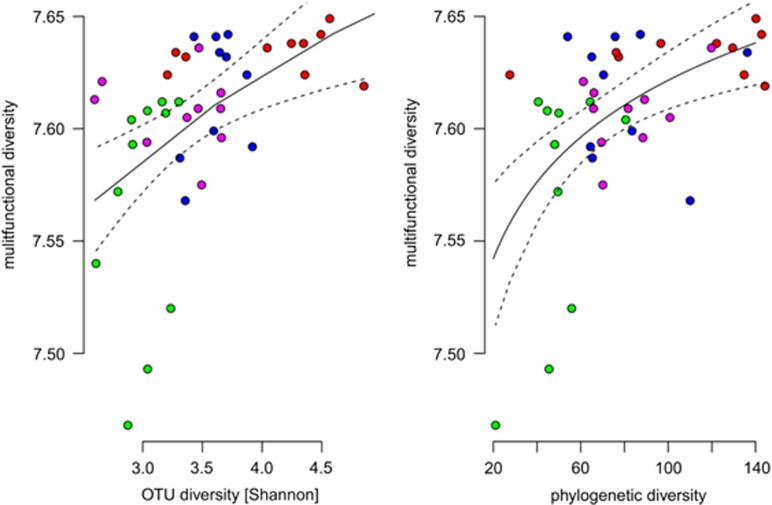
Accumulation curve showing the relationship between taxa diversity and PD with Shannon multifunctional diversity (based on all traits). Saturation indicates functional redundancy, which is higher when phylogenetic relationships are considered. Color codes are the same as in Figure 1.

To assess the potential functional consequences of shifts in bacterial communities along the turbidity gradient, we predicted multifunctional diversity using ancestral state reconstruction and metagenome prediction (Langille *et al.*, 2013; Miki *et al.*, 2013). The multifunctional diversity of traits associated to metabolism (*n*=146) and genetic information processing (*n*=28) were negatively related to turbidity (linear regression: *R*^2^=0.64, *P*=0.001 and *R*^2^=0.33, *P*=0.001, respectively; Figure 4). Diversity of unclassified traits (*n*=27), in contrast, was higher under more turbid conditions (linear regression: *R*^2^=0.57, *P*=0.001), whereas the diversity of traits associated with environmental information processing (*n*=28) was not significantly related to turbidity (linear regression: *R*^2^=0.04, *P*=0.11). Metagenomic surveys of glacier microbiomes are rare, however, a surprisingly large metabolic diversity was documented in glacial ice (Simon *et al.*, 2009), which may represent an adaptation to the low and heterogeneously distributed nutrients in glacier ice. The great diversity of traits associated with metabolic pathways in the clear lake may either indicate that the metabolic activity of microbes in turbid lakes is limited by less diverse substrates or may represent an adaptation to smaller seasonal variation in substrate availability. Both seem reasonable scenarios, as substrate diversity may be low in turbid lakes where inflow is dominated by glacier meltwater. The ecological role of traits involved in genetic information processing is mostly unclear, but the diversity of traits involved in DNA repair or folding of proteins may reflect adaptations to cope with stress caused by UV radiation in clear high mountain lakes. The large diversity of unclassified traits seemingly reflects the lack of knowledge of the functioning of high mountain lake bacterial communities in general and of proglacial lakes in particular. Functional redundancy or the ability of several taxa to perform the same function, may provide insurance against alteration in ecosystem functioning upon changes in community composition or loss of biodiversity (Miki *et al.*, 2013). However, accumulation curves of multifunctional diversity (including all traits) as a function of diversity suggested a limited buffering capacity for ecosystem functioning (Figure 5). Therefore, shifts in microbial diversity during the transition from turbid to clear lakes will likely result in a reduced multifunctional capacity.

### Conclusions and perspectives

In conclusion, climate change continues to threaten diversity and the multiple functions of communities in remote sentinel ecosystems such as proglacial lakes. Low-lying glaciers could disappear within short times (Zemp *et al.*, 2009; Vaughan *et al.*, 2013), and here we show that lake bacterial diversity adapted to the high turbidity and accompanying environmental conditions in these ecosystems is likely to change with glacier retreat. Glacier runoff is a source of diversity to turbid lakes, however, how different habitats on, within and below the glacier contribute to community assembly remains to be studied. Our analyses further indicate that ecosystem functioning, which encompasses the biogeochemical cycling of C, N and P, and biomass production, is also likely to shift during the transition between turbid and clear conditions. On a global scale, turbid glacial lakes seem a rather rare ecosystem type, however, as glaciers retreat, numerous new turbid lakes form depending on regional climatic and topographic conditions. In Switzerland, for instance, up to 500 new lakes larger than 1 ha and corresponding to a total lake area of 50–60 km^2^ may form (Linsbauer *et al.*, 2012), whereas in Central Asia, glacial lake area have significantly increased since 1968 (Mergili *et al.*, 2013). Upward shifts of species in combination with lake formation may provide short-term refuge for specialized communities with a yet unknown functional and genetic potential. However, once glaciers completely vanish, turbid lakes will rapidly shift to clear water conditions and specialized microbial diversity will be lost beyond retrieval.

## References

[bib1] Adrian R, O'Reilly CM, Zagarese H, Baines SB, Hessen DO, Keller W et al. (2009). Lakes as sentinels of climate change. Limnol Oceanogr 54: 2283–2297. 20396409 10.4319/lo.2009.54.6_part_2.2283 PMC2854826

[bib2] Anesio AM, Laybourn-Parry J. (2012). Glaciers and ice sheets as a biome. Trends Ecol Evol 27: 219–225. 22000675 10.1016/j.tree.2011.09.012

[bib3] Arrigo KR, van Dijken GL, Strong AL. (2015). Environmental controls of marine productivity hot spots around Antarctica. J Geophys Res Oceans 120: 5545–5565.

[bib4] Besemer K, Singer G, Quince C, Bertuzzo E, Sloan W, Battin TJ. (2013). Headwaters are critical reservoirs of microbial diversity for fluvial networks. Proc R Soc B Biol Sci 280: 20131760. 10.1098/rspb.2013.1760 PMC3790480 24089333

[bib5] Chen J, Bittinger K, Charlson ES, Hoffmann C, Lewis J, Wu GD et al. (2012). Associating microbiome composition with environmental covariates using generalized UniFrac distances. Bioinformatics 28: 2106–2113. 22711789 10.1093/bioinformatics/bts342 PMC3413390

[bib6] Crump BC, Amaral-Zettler LA, Kling GW. (2012). Microbial diversity in arctic freshwaters is structured by inoculation of microbes from soils. ISME J 6: 1629–1639. 22378536 10.1038/ismej.2012.9 PMC3498914

[bib7] DeSantis TZ, Hugenholtz P, Larsen N, Rojas M, Brodie EL, Keller K et al. (2006). Greengenes, a Chimera-Checked 16S rRNA Gene Database and Workbench Compatible with ARB. Appl Environ Microbiol 72: 5069–5072. 16820507 10.1128/AEM.03006-05 PMC1489311

[bib8] Egholm DL, Pedersen VK, Knudsen MF, Larsen NK. (2012). Coupling the flow of ice, water, and sediment in a glacial landscape evolution model. Geomorphology 141: 47–66.

[bib9] Fierer N, Hamady M, Lauber CL, Knight R. (2008). The influence of sex, handedness, and washing on the diversity of hand surface bacteria. Proc Natl Acad Sci USA 105: 17994–17999. 19004758 10.1073/pnas.0807920105 PMC2584711

[bib10] Freimann R, Buergmann H, Findlay SEG, Robinson CT. (2013). Bacterial structures and ecosystem functions in glaciated floodplains: contemporary states and potential future shifts. ISME J 7: 2361–2373. 23842653 10.1038/ismej.2013.114 PMC3834847

[bib11] Freimann R, Burgmann H, Findlay SEG, Robinson CT. (2014). Spatio-temporal patterns of major bacterial groups in alpine waters. PloS One 9: e113524. 25409508 10.1371/journal.pone.0113524 PMC4237416

[bib12] Gallegos CL, Davies-Colley RJ, Gall M. (2008). Optical closure in lakes with contrasting extremes of reflectance. Limnol Oceanogr 53: 2021–2034.

[bib13] Hood E, Berner L. (2009). Effects of changing glacial coverage on the physical and biogeochemical properties of coastal streams in southeastern Alaska. J Geophys Res Biogeosci 114: G03001.

[bib14] Hood E, Fellman J, Spencer RGM, Hernes PJ, Edwards R, D'Amore D et al. (2009). Glaciers as a source of ancient and labile organic matter to the marine environment. Nature 462: 1044–U1100. 20033045 10.1038/nature08580

[bib15] Jacobsen D, Milner AM, Brown LE, Dangles O. (2012). Biodiversity under threat in glacier-fed river systems. Nat Climate Change 2: 361–364.

[bib16] Kammerlander B, Breiner H-W, Filker S, Sommaruga R, Sonntag B, Stoeck T. (2015). High diversity of protistan plankton communities in remote high mountain lakes in the European Alps and the Himalayan mountains. FEMS Microbiol Ecol 91: fiv010. 25764458 10.1093/femsec/fiv010 PMC4399440

[bib17] Kembel SW, Cowan PD, Helmus MR, Cornwell WK, Morlon H, Ackerly DD et al. (2010). Picante: R tools for integrating phylogenies and ecology. Bioinformatics 26: 1463–1464. 20395285 10.1093/bioinformatics/btq166

[bib18] Knights D, Kuczynski J, Charlson ES, Zaneveld J, Mozer MC, Collman RG et al. (2011). Bayesian community-wide culture-independent microbial source tracking. Nat Methods 8: 761–U107. 21765408 10.1038/nmeth.1650 PMC3791591

[bib19] Koenings JP, Burkett RD, Edmundson JM. (1990). The exclusion of limnetic cladocerans from turbid glacier-meltwater lakes. Ecology 71: 57–67.

[bib20] Langille MGI, Zaneveld J, Caporaso JG, McDonald D, Knights D, Reyes JA et al. (2013). Predictive functional profiling of microbial communities using 16S rRNA marker gene sequences. Nat Biotech 31: 814–821. 10.1038/nbt.2676 PMC3819121 23975157

[bib21] Leibold MA, Holyoak M, Mouquet N, Amarasekare P, Chase JM, Hoopes MF et al. (2004). The metacommunity concept: a framework for multi-scale community ecology. Ecol Lett 7: 601–613.

[bib22] Linsbauer A, Paul F, Haeberli W. (2012). Modeling glacier thickness distribution and bed topography over entire mountain ranges with GlabTop: application of a fast and robust approach. J Geophys Res Earth Surface 117: F03007.

[bib23] Margesin R, Miteva V. (2011). Diversity and ecology of psychrophilic microorganisms. Res Microbiol 162: 346–361. 21187146 10.1016/j.resmic.2010.12.004

[bib24] Mergili M, Muller JP, Schneider JF. (2013). Spatio-temporal development of high-mountain lakes in the headwaters of the Amu Darya River (Central Asia). Global Planet Change 107: 13–24.

[bib25] Miki T, Yokokawa T, Matsui K. (2013). Biodiversity and multifunctionality in a microbial community: a novel theoretical approach to quantify functional redundancy. Proc R Soc B Biol Sci 281: 20132498. 10.1098/rspb.2013.2498 PMC3871314 24352945

[bib26] Milner AM, Brown LE, Hannah DM. (2009). Hydroecological response of river systems to shrinking glaciers. Hydrol Processes 23: 62–77.

[bib27] Oerlemans J. (2005). Extracting a climate signal from 169 glacier records. Science 308: 675–677. 15746388 10.1126/science.1107046

[bib28] Oksanen J, Blanchet FG, Kindt R, Legendre P, Minchin PR, O'Hara RB et al. (2013). vegan: Community Ecology Package. R package version 2.0-9. Available at: http://CRAN.R-project.org/package=vegan.

[bib29] Price MN, Dehal PS, Arkin AP. (2010). FastTree 2 – Approximately Maximum-Likelihood Trees for Large Alignments. PLoS ONE 5: e9490. 20224823 10.1371/journal.pone.0009490 PMC2835736

[bib30] R Development Core Team. (2013). *R: A Language and Environment for Statistical Computing*. R foundation for statistical computing: Vienna, Austria.

[bib31] Rose KC, Hamilton DP, Williamson CE, McBride CG, Fischer JM, Olson MH et al. (2014). Light attenuation characteristics of glacially-fed lakes. J Geophys Res Biogeosci 119: 1446–1457.

[bib32] Salcher MM, Pernthaler J, Psenner R, Posch T. (2005). Succession of bacterial grazing defense mechanisms against protistan predators in an experimental microbial community. Aquat Microb Ecol 38: 215–229.

[bib33] Scheffer M. (2003). Catastrophic regime shifts in ecosystems: linking theory to observation. Trends Ecol Evol 18: 648–656.

[bib34] Schloss PD, Gevers D, Westcott SL. (2011). Reducing the effects of PCR amplification and sequencing artifacts on 16S rRNA-based studies. Plos One 6: e27310. 22194782 10.1371/journal.pone.0027310 PMC3237409

[bib35] Segata N, Izard J, Waldron L, Gevers D, Miropolsky L, Garrett WS et al. (2011). Metagenomic biomarker discovery and explanation. Genome Biol 12: R60. 21702898 10.1186/gb-2011-12-6-r60 PMC3218848

[bib36] Simon C, Wiezer A, Strittmatter AW, Daniel R. (2009). Phylogenetic diversity and metabolic potential revealed in a glacier ice metagenome. Appl Environ Microbiol 75: 7519–7526. 19801459 10.1128/AEM.00946-09 PMC2786415

[bib37] Singer GA, Fasching C, Wilhelm L, Niggemann J, Steier P, Dittmar T et al. (2012). Biogeochemically diverse organic matter in Alpine glaciers and its downstream fate. Nat Geosci 5: 710–714.

[bib38] Slemmons KEH, Saros JE. (2012). Implications of nitrogen-rich glacial meltwater for phytoplankton diversity and productivity in alpine lakes. Limnol Oceanogr 57: 1651–1663.

[bib39] Slemmons KEH, Saros JE, Simon K. (2013). The influence of glacial meltwater on alpine aquatic ecosystems: a review. Environ Sci Processes Impacts 15: 1794–1806. 10.1039/c3em00243h 24056713

[bib40] Sommaruga R. (2015). When glaciers and ice sheets melt: consequences for planktonic organisms. J Plankton Res e-pub ahead of print 29 April 2015 doi:10.1093/plankt/fbv027. 10.1093/plankt/fbv027 PMC4747089 26869738

[bib41] Sommaruga R, Kandolf G. (2014). Negative consequences of glacial turbidity for the survival of freshwater planktonic heterotrophic flagellates. Sci Rep 4: 4113. 24531332 10.1038/srep04113 PMC3925964

[bib42] Stubbins A, Hood E, Raymond PA, Aiken GR, Sleighter RL, Hernes PJ et al. (2012). Anthropogenic aerosols as a source of ancient dissolved organic matter in glaciers. Nat Geosci 5: 198–201.

[bib43] Vaughan DG, Comiso JC, Allison I, Carrasco J, Kaser G, Kwok R et al. (2013) Observations: cryosphere. In: Stocker TF, Qin D, Plattner G-K, Tignor M, Allen SK, Boschung J, Nauels A, Xia Y, Bex V, Midgley PM (eds). Climate Change 2013: The Physical Science Basis. Contribution of Working Group I to the Fifth Assessment Report of the Intergovernmental Panel on Climate Change. Cambridge University Press: Cambridge, UK and New York, NY, USA.

[bib44] Wilhelm L, Singer GA, Fasching C, Battin TJ, Besemer K. (2013). Microbial biodiversity in glacier-fed streams. ISME J 7: 1651–1660. 23486246 10.1038/ismej.2013.44 PMC3721114

[bib45] Zemp M, Hoelzle M, Haeberli W. (2009). Six decades of glacier mass-balance observations: a review of the worldwide monitoring network. Ann Glaciol 50: 101–111.

