# Supplementary material for: Shifts in diversity and function of lake bacterial communities upon glacier retreat
Source: ISME J. 2016 Jan 15;10(7):1545–54. doi: 10.1038/ismej.2015.245 (PMC4852812; doi:10.1038/ismej.2015.245)
Supplement: Supplementary Information [file ismej2015245x1.doc]

**Supporting Material 1**

Panel A provides a map of the Faselfad catchment with the glacier, the uppermost lake FAS 1 (2600 m a.s.l.), the turbid lakes FAS 3 (2400 m a.s.l) and FAS 6 (2200 m a.s.l.) and the clear lake FAS4 (2400 m a.s.l.). FAS 1 and FAS 3 are connected with a stream (indicated by dashed lines), however, depending on time of the year this stream flows partially below the surface. FAS 4 and FAS 6 are also connected by a partially subsurface stream. Panel B shows an orthophotography of the same area. Panel C – F provide photographs taken from the shores of the lakes during the first sampling campaign (see inserts for location and date). Photography courtesy of F. Drewes.

A

B

glacier

FAS 1

FAS 3

FAS 4

FAS 6

Map source: <http://www.tirol.gv.at/tiris>


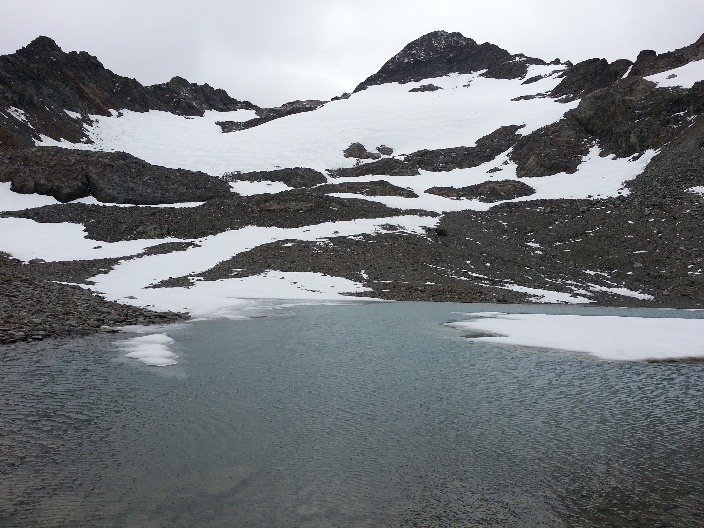

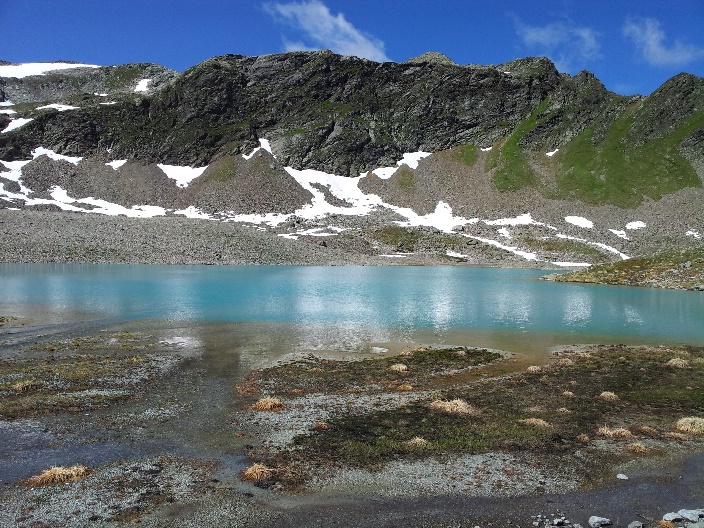


C

D

E

F

FAS 1 17.07.2012

FAS 3 18.07.2012


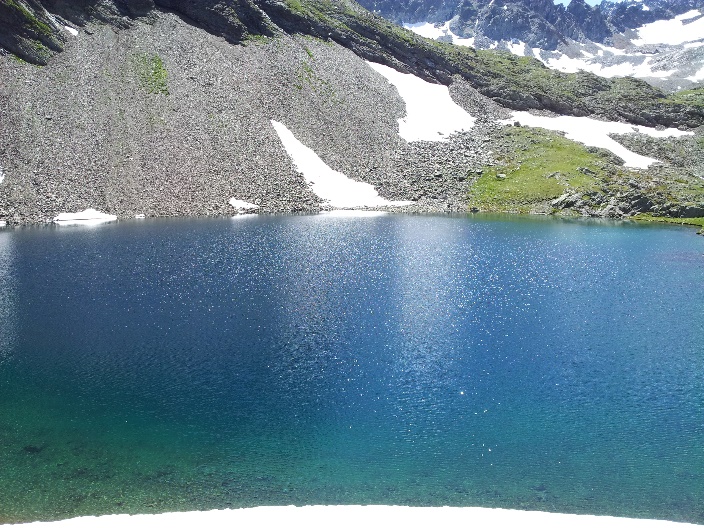

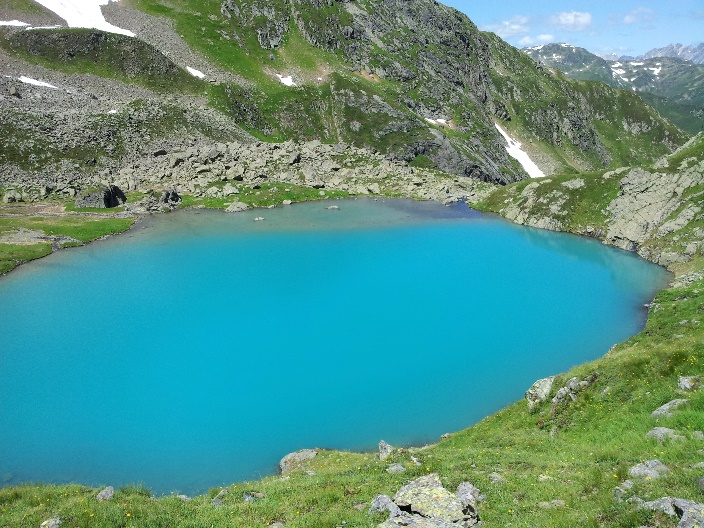


FAS 4 18.07.2012

FAS 6 18.07.2012
