# Supplementary material for: Shifts in diversity and function of lake bacterial communities upon glacier retreat
Source: ISME J. 2016 Jan 15;10(7):1545–54. doi: 10.1038/ismej.2015.245 (PMC4852812; doi:10.1038/ismej.2015.245)
Supplement: Supplementary Table 1 [file ismej2015245x2.doc]

**Supporting Table 1** Key environmental parameters, chlorophyll-a concentrations and bacterial cell numbers of the Faselfad lakes during the ice-free season of 2012.

| site | date | Turbidity  [NTU] | DOC  [µg L^-1^] | TDP  [µg L^-1^] | DN  [µg L^-1^] | Chlorophyll-a  [µg L^-1^] | Bacterial abundance  [10^5^ cells mL^-1^] |
| --- | --- | --- | --- | --- | --- | --- | --- |
| FAS 1 | 07/17/12 | 13.3 | 198.0 | 2.3 | 161.3 | 0.12 | 0.68 |
|  | 08/01/12 | 11.6 | 176.0 | 3.5 | 149.5 | 0.13 | 1.48 |
|  | 08/28/12 | 42.8 | 267.2 | 4.6 | 194.5 | 0.13 | 2.27 |
|  | 10/02/12 | 6.2 | 137.2 | 2.5 | 254.7 | NA | 1.59 |
| FAS 3 | 07/17/12 | 9.9 | 218.3 | 4.0 | 162.0 | 0.99 | 1.46 |
|  | 08/01/12 | 3.4 | 174.4 | 1.5 | 141.2 | 1.14 | 6.10 |
|  | 08/28/12 | 11.2 | 412.2 | 2.5 | 137.8 | 4.57 | 3.89 |
|  | 10/02/12 | 4.6 | 212.5 | 1.5 | 189.2 | NA | 2.05 |
| FAS 4 | 07/17/12 | 0.2 | 219.3 | 0.7 | 191.3 | 0.37 | 1.52 |
|  | 08/01/12 | 0.3 | 241.7 | 0.9 | 175.1 | 1.15 | 1.02 |
|  | 08/28/12 | 0.2 | 373.0 | 2.0 | 171.0 | 1.21 | 3.03 |
|  | 10/02/12 | 0.1 | 319.6 | 0.6 | 176.4 | NA | 0.79 |
| FAS 6 | 07/17/12 | 1.3 | 253.0 | 1.0 | 139.0 | 0.47 | 1.86 |
|  | 08/01/12 | 2.4 | 281.3 | 1.5 | 136.0 | 0.45 | 4.10 |
|  | 08/28/12 | 6.3 | 339.8 | 1.3 | 147.2 | 4.36 | 2.70 |
|  | 10/02/12 | 2.4 | 295.7 | 1.1 | 160.4 | NA | 1.77 |
