# Supplementary material for: Shifts in diversity and function of lake bacterial communities upon glacier retreat
Source: ISME J. 2016 Jan 15;10(7):1545–54. doi: 10.1038/ismej.2015.245 (PMC4852812; doi:10.1038/ismej.2015.245)
Supplement: Supplementary Table 2 [file ismej2015245x3.doc]

**Supporting Table 2** Non-exhaustive literature search for Nitrospirae in glacial, cold or alpine environments.

| **environment** | **location** | **reference** |
| --- | --- | --- |
| proglacial stream | glacier runoff, Austria | (1) |
| glacial lake shore soil | Roopkund glacier, Himalaya, India | (2) |
| glacial ice | Laohugou Glacier No. 12, China | (3) |
| proglacial soil | Kafni glacier, Himalayan, India | (4) |
| proglacial soil | Pindari glacier, Himalaya, India | (5) |
| proglacial soil | Tianshan glacier, China | (6) |
| proglacial soil | West Spitsbergen, Norway | (7) |
| proglacial soil | Broknes Peninsula, Antarctica | (8) |

1. Wilhelm L, Singer GA, Fasching C, Battin TJ, & Besemer K (2013) Microbial biodiversity in glacier-fed streams. *ISME J* 7(8):1651-1660.

2. Pradhan S*, et al.* (2010) Bacterial biodiversity from Roopkund Glacier, Himalayan mountain ranges, India. *Extremophiles* 14(4):377-395.

3. Zhang S*, et al.* (2015) Preliminary Study on Effects of Glacial Retreat on the Dominant Glacial Snow Bacteria in Laohugou Glacier No. 12. *Geomicrobiology Journal* 32(2):113-118.

4. Srinivas TNR*, et al.* (2011) Comparison of bacterial diversity in proglacial soil from Kafni Glacier, Himalayan Mountain ranges, India, with the bacterial diversity of other glaciers in the world. *Extremophiles* 15(6):673-690.

5. Shivaji S*, et al.* (2011) Bacterial diversity of soil in the vicinity of Pindari glacier, Himalayan mountain ranges, India, using culturable bacteria and soil 16S rRNA gene clones. *Extremophiles* 15(1):1-22.

6. Wu X*, et al.* (2012) Bacterial diversity in the foreland of the Tianshan No. 1 glacier, China. *Environmental Research Letters* 7(1).

7. Schuette UME*, et al.* (2010) Bacterial diversity in a glacier foreland of the high Arctic. *Molecular Ecology* 19:54-66.

8. Bajerski F & Wagner D (2013) Bacterial succession in Antarctic soils of two glacier forefields on Larsemann Hills, East Antarctica. *Fems Microbiology Ecology* 85(1):128-142.
