# Supplementary material for: Shifts in diversity and function of lake bacterial communities upon glacier retreat
Source: ISME J. 2016 Jan 15;10(7):1545–54. doi: 10.1038/ismej.2015.245 (PMC4852812; doi:10.1038/ismej.2015.245)
Supplement: Supplementary Figure 1 [file ismej2015245x4.pdf]

Supporting Figure 1 Distribution of OTUs among turbid and clear lakes and glacier runoff samples. A ternary plot showing mean relative abundance of each OTU in samples of turbid and clear alpine lakes and the glacier runoff (**panel A**). The colors indicate phylogenetic affiliation, circle size reflects relative abundance (log-transformed). OTUs equally common in the three sample categories are located in the center of the ternary plot. Abundant OTUs (large circles) were found in similar relative abundance in the three categories and mainly belonged to the beta-subclass of Proteobacteria (see also **panel B** for a plot of occupancy versus total sequence abundance for each OTU). Most of the OTUs were either exclusively found in the turbid lakes and glacier runoff or shared among different categories, whereas only few OTUs were shared between the glacier runoff and the clear lake, highlighting the reluctance of glacier-associated microbes to establish in clear alpine lakes. **Panel C** provides a heatmap of relative abundance (log transformed, see color key) of the 60 most abundant OTUs in all samples. The highest taxonomic classification is indicated along with the OTU number. Note the high relative abundance of several OTUs in the glacier and FAS1, which became less abundant in the turbid lakes FAS3 and FAS6. A group of OTUs related to Bacteroidetes occured in high relative abundance in the clear lake FAS4, however was absent in the turbid lakes and the glacier runoff.

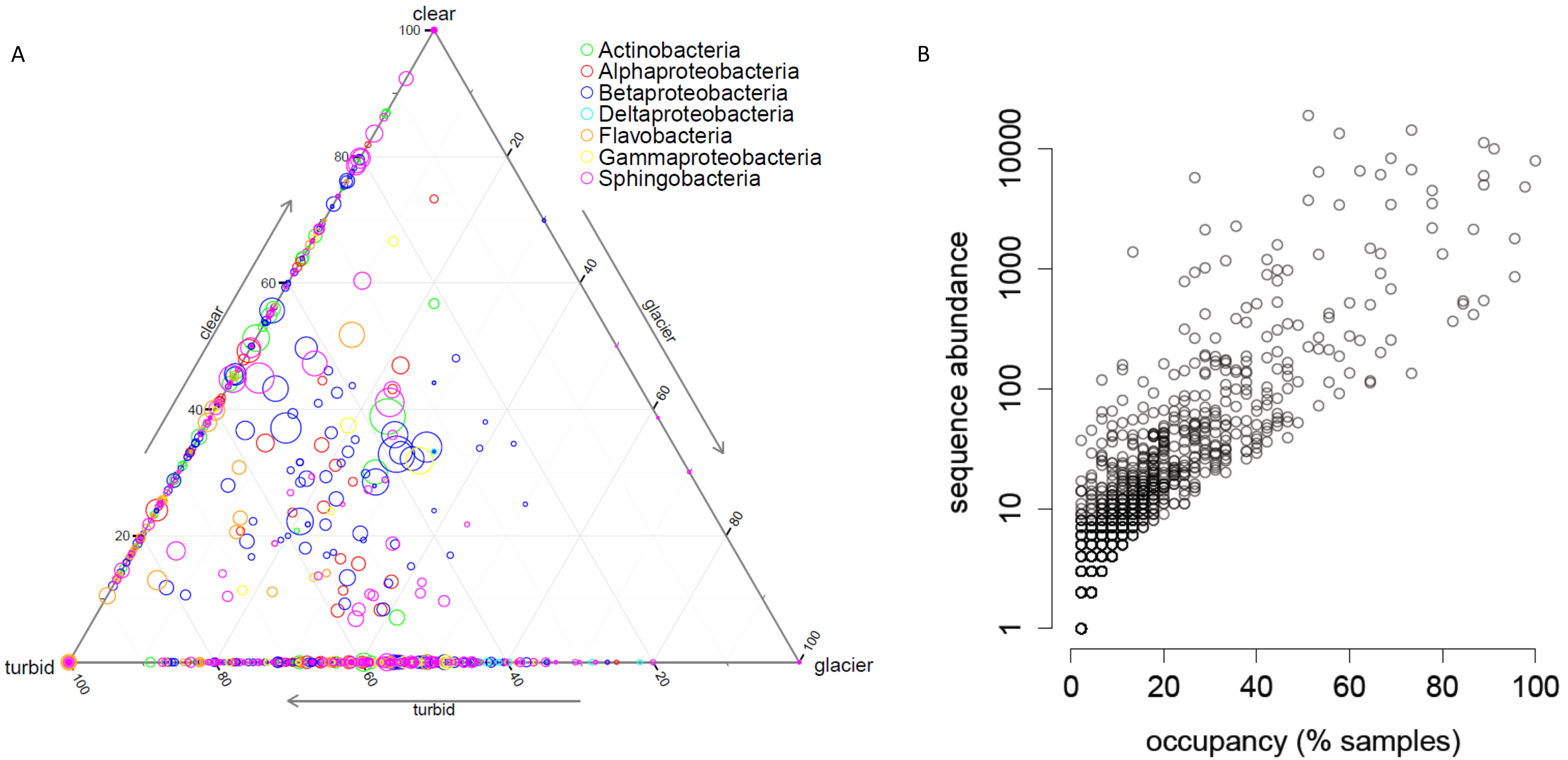

C

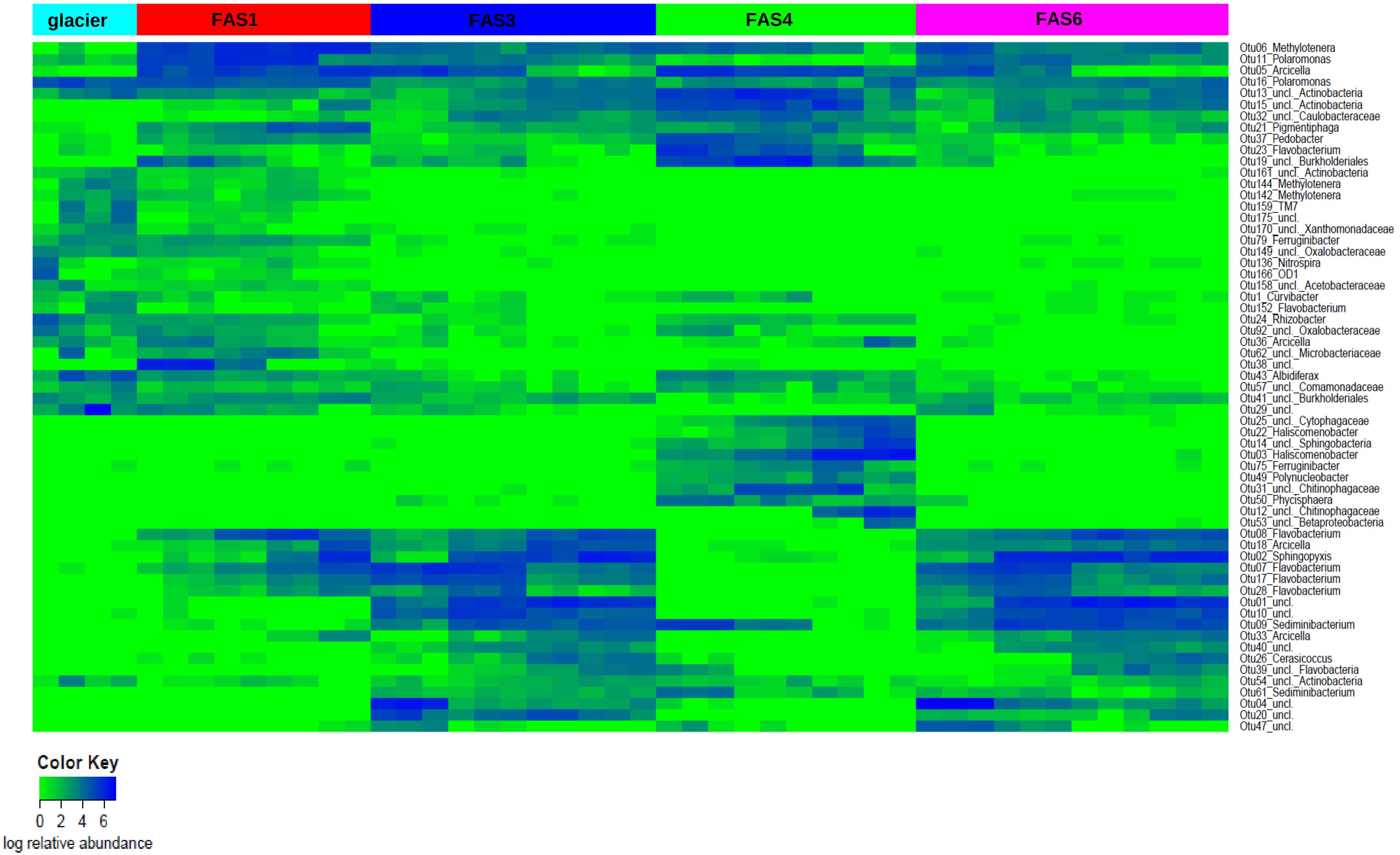

glacier
FAS1
FAS3
FAS4
FAS6
Otu06_Methylotenera
Otu11_Polaromonas
Otu05_Arcicella
Otu16_Polaromonas
Otu13_uncl._Actinobacteria
Otu15_uncl._Actinobacteria
Otu32_uncl._Caulobacteraceae
Otu21_Pigmentiphaga
Otu37_Pedobacter
Otu23_Flavobacterium
Otu19_uncl._Burkholderiales
Otu161_uncl._Actinobacteria
Otu144_Methylotenera
Otu142_Methylotenera
Otu159_TM7
Otu175_uncl.
Otu170_uncl._Xanthomonadaceae
Otu79_Ferruginibacter
Otu149_uncl._Oxalobacteraceae
Otu136_Nitrospira
Otu166_OD1
Otu158_uncl._Acetobacteraceae
Otu1_Curvibacter
Otu152_Flavobacterium
Otu24_Rhizobacter
Otu92_uncl._Oxalobacteraceae
Otu36_Arcicella
Otu62_uncl._Microbacteriaceae
Otu38_uncl.
Otu43_Albidiferax
Otu57_uncl._Comamonadaceae
Otu41_uncl._Burkholderiales
Otu29_uncl.
Otu25_uncl._Cytophagaceae
Otu22_Haliscomenobacter
Otu14_uncl._Sphingobacteria
Otu03_Haliscomenobacter
Otu75_Ferruginibacter
Otu49_Polynucleobacter
Otu31_uncl._Chitinophagaceae
Otu50_Phycisphaera
Otu12_uncl._Chitinophagaceae
Otu53_uncl._Betaproteobacteria
Otu08_Flavobacterium
Otu18_Arcicella
Otu02_Sphingopyxis
Otu07_Flavobacterium
Otu17_Flavobacterium
Otu28_Flavobacterium
Otu01_uncl.
Otu10_uncl.
Otu09_Sediminibacterium
Otu33_Arcicella
Otu40_uncl.
Otu26_Cerasicoccus
Otu39_uncl._Flavobacteria
Otu54_uncl._Actinobacteria
Otu61_Sediminibacterium
Otu04_uncl.
Otu20_uncl.
Otu47_uncl.
Color Key
0 2 4 6
log relative abundance
