# Supplementary figures and images for: Shifts in diversity and function of lake bacterial communities upon glacier retreat
Source: ISME J. 2016 Jan 15;10(7):1545–54. doi: 10.1038/ismej.2015.245 (PMC4852812; doi:10.1038/ismej.2015.245)

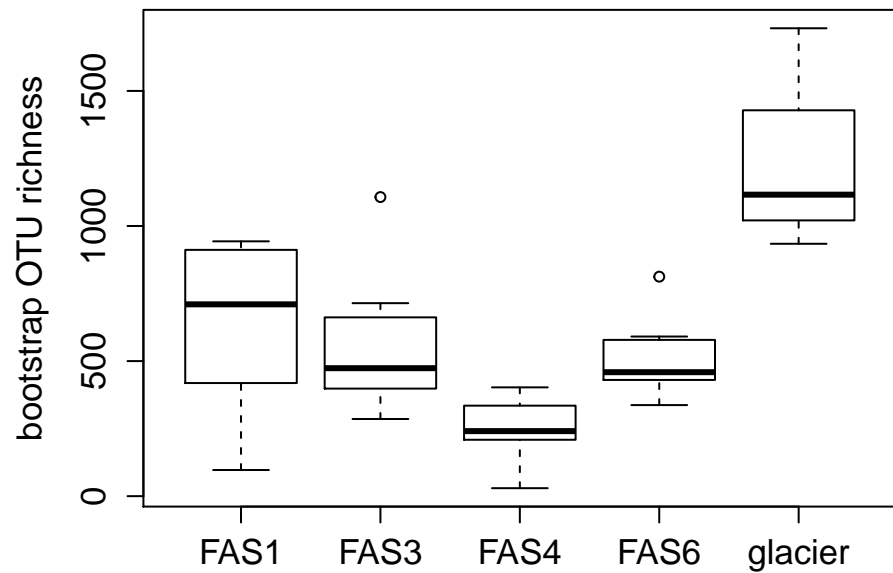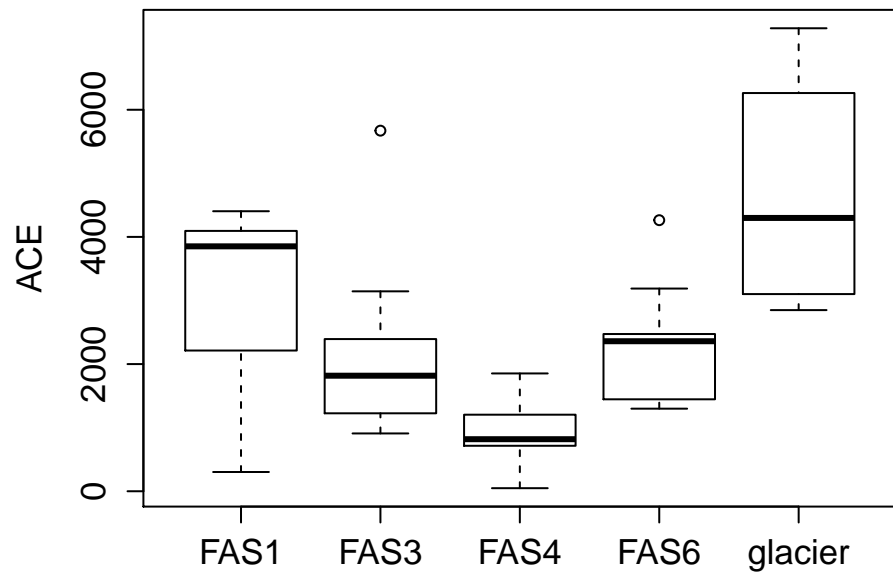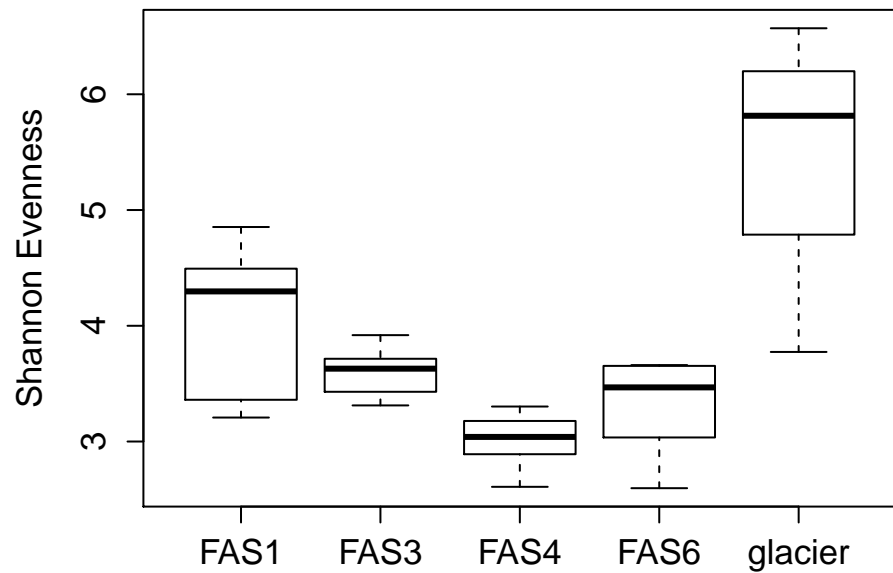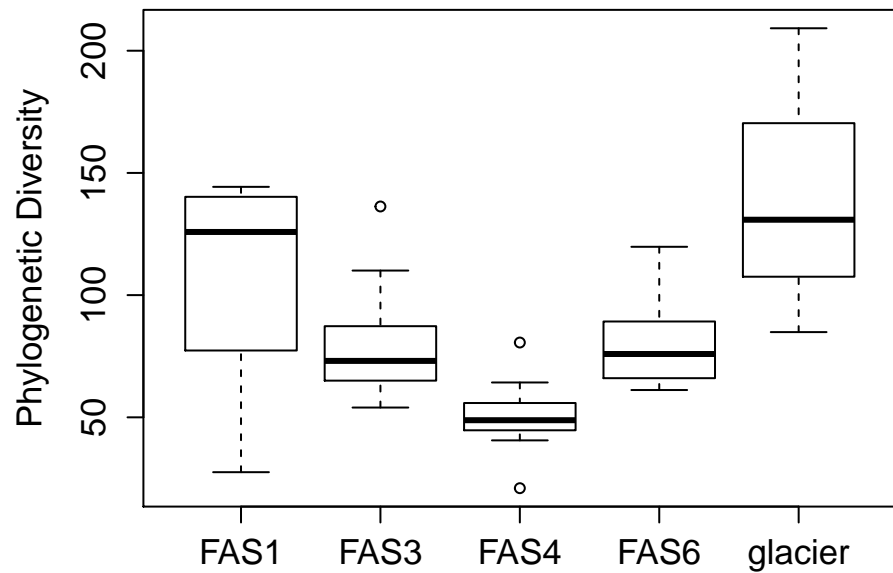

Supplement: Supplementary Figure 2 [file ismej2015245x5.pdf]

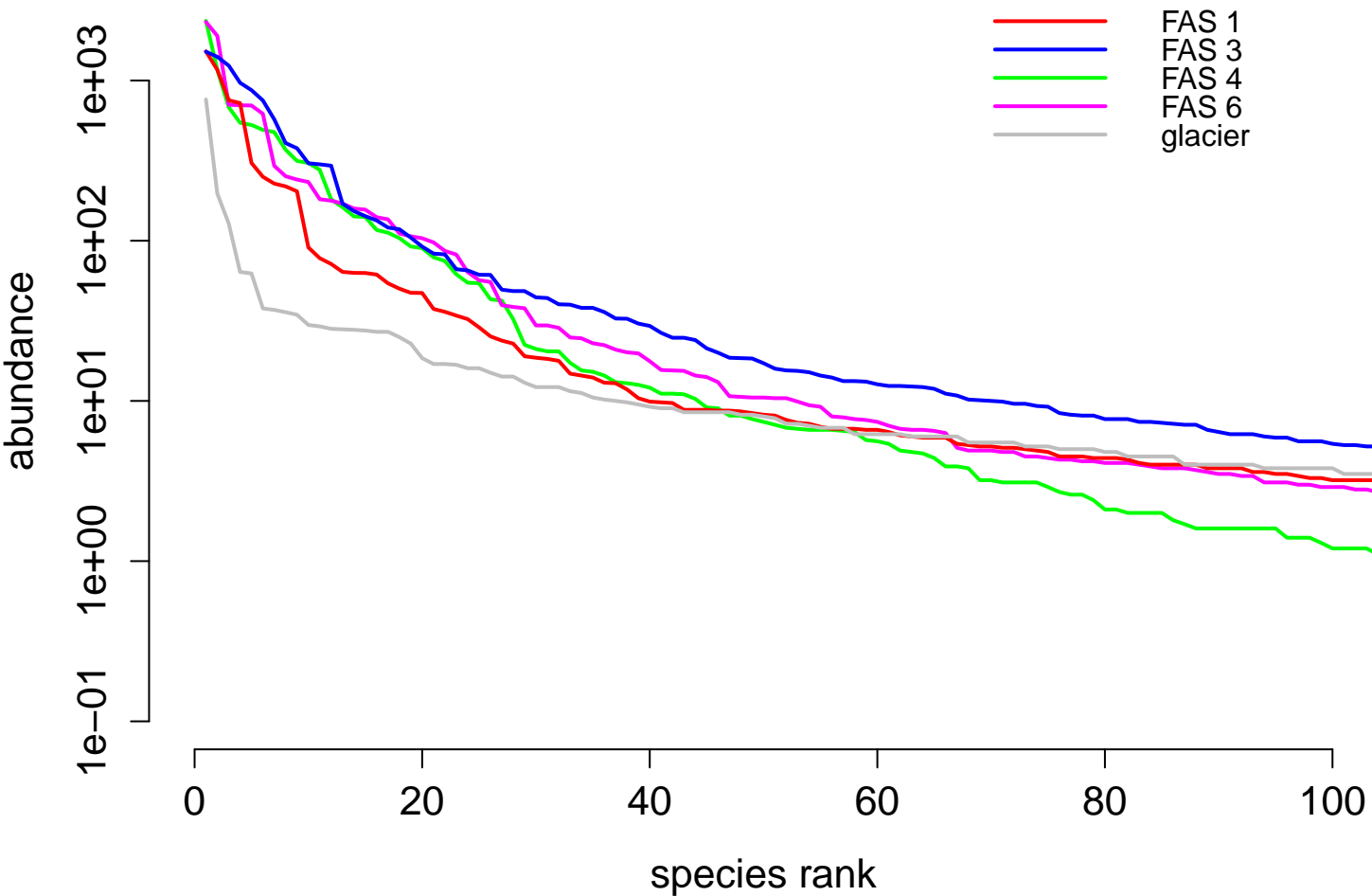

Supplement: Supplementary Figure 3 [file ismej2015245x6.pdf]
